# Anti-EGFR Indocyanine Green-Mitomycin C-Loaded Perfluorocarbon Double Nanoemulsion: A Novel Nanostructure for Targeted Photochemotherapy of Bladder Cancer Cells

**DOI:** 10.3390/nano8050283

**Published:** 2018-04-26

**Authors:** Yu-Hsiang Lee, Yu-Chun Lin

**Affiliations:** 1 Department of Biomedical Sciences and Engineering, National Central University. No. 300, Jhongda Rd., Taoyuan City 32001, Taiwan; carlo12lin@yahoo.com.tw; 2 Department of Chemical and Materials Engineering, National Central University, Taoyuan City 32001, Taiwan

**Keywords:** bladder cancer, EGFR, photochemotherapy, indocyanine green, mitomycin C, perfluorocarbon, double emulsion

## Abstract

The use of phototherapy as an adjuvant bladder cancer treatment has long been considered, but its application has been severely hampered due to a lack of tumor specificity, unpredicted cytotoxicity, and insufficient anticancer efficacy. In this study, we aim to manufacture anti-EGFR indocyanine green (ICG) mitomycin C (MMC) encapsulated perfluorocarbon double nanoemulsions (EIMPDNEs), and explore their photochemotherapeutic efficacy on EGFR-expressing bladder cancer cells in vitro. The EIMPDNEs were manufactured using a double emulsification technique followed by antibody conjugation on the particles’ surfaces. The EIMPDNE were 257 ± 19.4 nm in size, with a surface charge of −12.3 ± 2.33 mV. The EGFR targetability of the EIMPNDE was confirmed by its enhanced binding efficiency to T24 cells when compared with the performance of nanodroplets without EGFR conjugation (*p* < 0.05). In comparison with freely dissolved ICG, the EIMPDNEs with equal ICG content conferred an improved thermal stability to the encapsulated ICG, and were able to provide a comparable hyperthermia effect and significantly enhanced the production of singlet oxygen under 808 nm near infrared (NIR) exposure with an intensity of 6 W cm^−2^ for 5 min (*p* < 0.05). Based on viability analyses, our data showed that the EIMPDNEs were effective in bladder cancer cell eradication upon NIR exposure (808 nm; 6 W cm^−2^), and the resulting cell death rate was even higher than that caused by a five-fold higher amount of entrapped MMC alone. With the merits of improved ICG stability, EGFR binding specificity, and effective cancer cell eradication, the EIMPDNEs exhibit potential for use in EGFR-expressing bladder cancer therapy with lower chemotoxicity.

## 1. Introduction

Clinically, urothelial cell carcinoma (UCC) remains one of the most fatal diseases of the urological system, of which bladder cancer—a lower urinary tract cancer—is the most frequently diagnosed, accounting for a very high number of deaths each year [1]. According to the statistics of the World Health Organization, bladder cancer ranks as the ninth most frequently diagnosed cancer, with a higher incidence in men, and it is the 13th leading cause of death from cancer worldwide [2]. In addition to its prevalence, bladder cancer is the most expensive malignancy to treat from diagnosis to death [3]. In general, patients with bladder cancer can be classified into two histopathological stages: Approximately 70% of cases are confined to the mucosa with no damage to the basement membrane (non-muscle-invasive bladder cancer, or NMIBC; stage Ta), whereas the other 30% present with an invasive cancer that may breach the basement membrane (T1) or even muscle (muscle-invasive bladder cancer, or MIBC; stage T2–T4), and may subsequently experience unpredictable metastasis. Currently, MIBC remains difficult to cure, and the five-year overall survival rate is still ≤50% after radical cystectomy, radiotherapy, and/or transurethral resection [4]. Although more than two-thirds of newly diagnosed bladder cancers are superficial/NMIBC, these cases are usually at high risk of recurrence (50%–70%) [5], and for those who experience recurrence, 20%–40% of cases may progress to MIBC and thereafter become subject to ineffective therapeutics [6]. These circumstances indicate that an effective strategy for NMIBC therapy is still urgently needed.

The epidermal growth factor receptor (EGFR; also known as ErbB1) is a member of the tyrosine kinase receptor family—a group of receptors which are all encoded by the c-ErbB oncogenes—and these proto-oncogenes are associated with the interference of cell growth regulation, increased angiogenesis, and reduced apoptosis that typifies malignancy [7,8,9]. In terms of UCC, EGFR is overexpressed in up to 74% of bladder cancer cells [10], but has a relatively low expression level in normal urothelium [11]. Moreover, the expression level of EGFR is highly associated with tumor grade, stage progression, and poor therapeutic outcome in bladder cancer, as reported in numerous studies [12,13,14]. In addition, EGFR is localized to the basal layer of urothelial cells in normal urothelium, but is extensively present in both the luminal and basal layers in UCC [15], indicating that an EGFR-target intravesical treatment has potential for use in bladder cancer cell eradication. Overall, this shows that the EGFR appears to be a feasible target for bladder cancer/NMIBC therapy. 

Mitomycin C (MMC) is one of the United States (US) FDA-approved chemotherapeutic drugs, and its antitumor efficacy is achieved through DNA cross-linking/breaking [16], glutathione alkylation [17], and inhibition of thioredoxin reductase [18]. Although MMC has been widely used in neoplastic diseases, the dose of MMC utilized in the clinic is still of concern due to detrimental side effects, such as cystitis [19] and skin eruptions [20]. To circumvent these issues, the co-administration of antitumor agents or methods is frequently considered as a potential regimen for cancer treatment because it may help to decrease the effective dosage of the drug needed, thereby reducing its potential chemotoxicity and leading to an improved therapeutic outcome. Among the various anticancer strategies, near infrared (NIR)-based phototherapy has received increasing attention as an adjuvant to bladder cancer therapy because it may provide (1) less toxicity to normal cells/tissues through the use of targeted photosensitive agents and/or a spatially controlled light irradiating operation, (2) enhanced tissue penetration effectiveness when compared with that achieved by UV/visible light, and (3) increased cell membrane permeability for drug delivery [21,22]. In general, phototherapy is functionalized by induced reactive oxygen species (ROS) generated from the photosensitizers under light illumination in the presence of oxygen and/or its hyperthermia effect. The former is able to interfere with cellular metabolism and thereby trigger programmed cell death (i.e., photodynamic therapy (PDT)), whereas the latter may cause thermal ablation of cancer cells—this is known as photothermal therapy (PTT) [22,23,24]. However, no matter which mechanism is preferred, the photosensitizer agent plays a key role in the effect of phototherapy.

Indocyanine green (ICG), which is a type of water-soluble tricarbocyanine dye, is one of the only clinically approved NIR fluorophores, along with methylene blue. So far, in addition to serving as a fluorescent agent for many diagnostic applications, such as optical coherence tomography-angiography [25] and NIR fluorescence-guided oncologic surgery [26], ICG has been widely utilized for neoplastic phototherapy, including for skin, brain, and breast tumors [27,28,29]. This is due to its performance in heat and singlet oxygen generation upon NIR treatment. However, several drawbacks of ICG, such as rapid plasma clearance [30] and high aqueous degradability [31], seriously hamper its applicability in the clinic. 

Nanotechnology or nanomaterial may provide a feasible means for the simultaneous use of multi-agents such as MMC and ICG without the aforementioned disadvantages, because it may offer increased security, improved stability, and enhanced bioavailability of the payloads [32]. In this study, we aim to develop and manufacture a type of anti-EGFR ICG-MMC-encapsulated perfluorocarbon (PFC) double nanoemulsion (EIMPDNE) to explore the impact of joint photo and chemotherapeutics on EGFR-expressing bladder cancer cells. PFC, which is a fluorine-substituted derivative of hydrocarbons, is a well-known robust oxygen transporter since it can dissolve much larger respiratory gases than water (O_2_ and CO_2_) [33]. Such a characteristic implies that the PFC constituent will be advantageous for EIMPDNEs in terms of their use in PDT. We anticipate that the developed EIMPDNEs are able to (1) effectively protect the encapsulated ICG from aqueous degradation that is caused by external stimuli, such as pH, light, and/or heat [30,34], (2) possess EGFR binding specificity with EGFR-expressing bladder cancer cells to reduce off-target cytotoxicity caused by MMC, and (3) provide effective cancer cell eradication with reduced chemotoxicity, because multiplex photochemotherapeutics may decrease the efficacious dose of the anticancer drug when compared with the dose that is required in chemotherapy alone. In this paper, we first introduce the manufacturing processes of the EIMPDNEs, followed by investigating their physicochemical properties, functionalities, and anticancer efficacy.

## 2. Materials and Methods

### 2.1. Fabrication of EIMPDNEs

**Figure 1:**
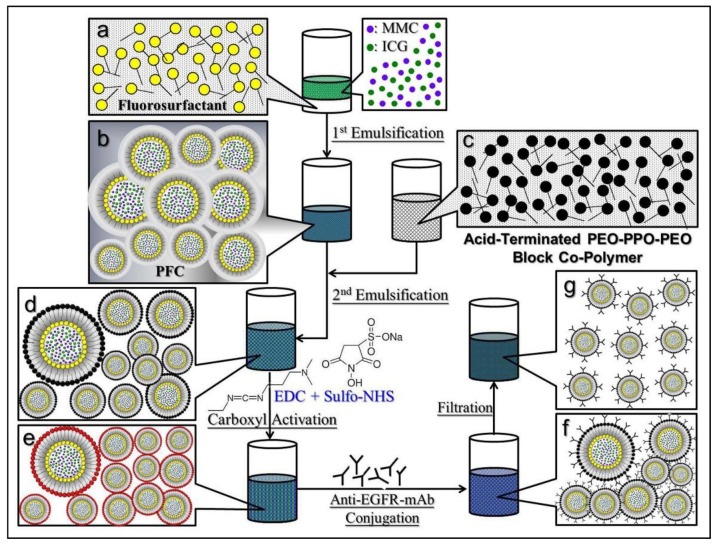
Schematic illustration of the manufacture protocol of the encapsulated perfluorocarbon double nanoemulsions (EIMPDNEs). The IMPDNEs structured with fluorosurfactant and acid-terminated PEO-PPO-PEO block co-polymer were first formed by using a modified emulsification approach (**a** → **d**), followed by *N*-(3-dimethylaminopropyl)-*N*’-ethylcarbodiimide hydrochloride (EDC)/sulfo-NHS-mediated carboxylic activation (**e**) and anti-epidermal growth factor receptor (EGFR)-mAb conjugation (**f**) on the nanoemulsion surface to accomplish the EIMPDNE formation. To remove unreacted/excessive chemicals and simultaneously narrow down the size dispersity of the nanodroplets, the produced EIMPDNEs were subjected to a filtration process using a 0.45 µm filter and displayed an improved size uniformity thereafter (**g**).

The ICG-MMC-encapsulated PFC double nanoemulsions (IMPDNEs) were first fabricated by using a modified emulsification approach. Briefly, polyethoxylated fluorosurfactant was first dissolved in perfluorooctyl bromide (PFOB; Sigma-Aldrich, St. Louis, MO, USA) to form a surfactant-PFC solution (1% (*w*/*v*)). Next, 550 µL of methanol (50% (*v*/*v*)) containing ICG (Sigma-Aldrich) and MMC (Selleckchem, Houston, TX, USA) (0.1% (*w*/*v*) for each) was slowly added to the surfactant-PFC solution. The primary water-in-PFC (W1/PFC) emulsions were obtained after the mixture was sonicated for 10 min under an ice bath. The primary emulsions were then slowly added into a PBS containing acid-terminated PEO-PPO-PEO block copolymer (carboxylic Pluronic^®^ F68; 5% *w*/*w*), which was synthesized according to the previous report [35], followed by a 10 min sonication to obtain the W1/PFC/W2 double emulsions (the IMPDNEs). After being washed twice with PBS, the IMPDNEs were immediately subjected to anti-EGFR-monoclonal antibody (mAb) (Cell signaling, Danvers, MA, USA) conjugation on the droplet surface through the carboxyl-amine crosslinking reaction. Briefly, the IMPDNEs were first reacted with *N*-(3-dimethylaminopropyl)-*N*’-ethylcarbodiimide hydrochloride (EDC; Sigma-Aldrich) and sulfo-*N*-hydroxysuccinimide (Sulfo-NHS; Sigma-Aldrich) (with a molar ratio of EDC/sulfo-NHS = 9:1) in PBS under an ambient temperature for 2 h. After being washed twice with PBS, the carboxyl-activated nanodroplets were mixed with 100 µg of anti-EGFR-mAbs in a total of 1 mL PBS, and was maintained at room temperature in the dark for 1 h. To remove excess/unreacted molecules and simultaneously reduce the size dispersity of the products, the yielded nanodroplets (EIMPDNEs) were washed twice with deionized (DI) water and were subsequently filtrated through a 0.45 μm filter. The harvested EIMPDNEs were then lyophilized for 24 h and were stored at 4 °C for further use. The overall procedures of the EIMPDNE manufacture are illustrated in Figure 1. 

### 2.2. Verification of Anti-EGFR-mAb Conjugation

The presence and bioactivity of anti-EGFR-mAbs on the surface of the EIMPDNE were determined by using the fluorescent anti-mouse immunoglobulin G (IgG) secondary mAb (FS-mAb; Cell signaling) as the probe. The fluorescence expressed by the FS-mAb-treated nanoemulsions was detected through both fluorescent microscopy and spectrofluorometry performed with an excitation wavelength of 488 nm and emission wavelength of 525 nm. In this study, the fluorescence level was quantitatively represented by relative fluorescence units (RFUs) and it was analyzed after normalization against the background signal.

### 2.3. Assessments of the Physical and Chemical Properties of the EIMPDNEs

The surface charge (ζ-potential) and the size distribution of the EIMPDNEs were determined through the measurement technique of dynamic light scattering (DLS, Zetasizer Nano-ZS90, Malvern, Worcestershire, UK). The morphology of the EIMPDNEs was detected using a scanning electron microscope (SEM, Philips XL-30 FEG, Royal Philips Electronics, Amsterdam, The Netherlands). The encapsulation rates (*R*_E_) of ICG and MMC for the EIMPDNE were calculated by Equation (1):

(1) $$R_{E}=\frac{W_{0}-W_{\sup}}{W_{0}}\times 100\%,$$

where *W*_sup_ denotes the amount of unencapsulated drug molecules found in the bulk phase of the sample and *W*_0_ is the total amount of ICG or MMC initially used for the EIMPDNE fabrication. Both *W*_sup_ and *W*_0_ were determined by using a spectrophotometer (V-650, JASCO, Easton, MD, USA) with λ_abs_ = 780 nm for ICG and 367 nm for MMC based on Beer-Lambert’s law. The loading rates of ICG and MMC in the EIMPDNE (*R*_L_; wt %) were calculated by Equation (2):

(2) $$R_{L}=\frac{W_{d}}{W_{\mathrm{NE}}}\times 100\%,$$

where *W*_NE_ is the weight of the EIMPDNE sample and *W*_d_ denotes the weight of the ICG or the MMC entrapped in the nanodroplet (~*W*_0_ × *R*_E_).

### 2.4. Analysis of EIMPDNE Stability

The degradation of the EIMPDNE-entrapped ICG and the release kinetics of the entrapped MMC at 4 and 37 °C were evaluated immediately after the EIMPDNEs were fabricated. All of the EIMPDNE samples were wrapped in foil to prevent light illumination/photodegradation throughout the experiment. After treatment for 2, 4, 12, 24, and 48 h, the EIMPDNEs and their supernatant harvested post centrifugation were separately detected by using a spectrophotometer set at λ_abs_ = 780 nm for the EIMPDNE samples and λ_abs_ = 367 nm for the supernatant, in order to analyze the residual amount of ICG in the nanodroplets and the quantity of MMC released to the bulk phase, respectively. In this study, the degradation rate coefficient (*k*_d_) of ICG in each group was determined based on the dynamic method [25]:

(3) $$\frac{C_{t}}{C_{0}}=\exp(-k_{d}\times t),$$

where *C*_0_ and *C*_t_ represent the concentrations of ICG in the matrix (EIMPDNE or DI water) at time *t* = 0 and a specific time *t* > 0, respectively. The cumulative release rate of MMC (*CR*_M_) at each time point was evaluated by Equation (4):

(4) $$CR_{M}=\frac{W_{\mathrm{Mt}}}{W_{M0}^{}}\times 100\%,$$

where *W*_M0_ means the amount of MMC in the EIMPDNE sample in the beginning (*t* = 0), and *W*_Mt_ denotes the amount of MMC obtained in the supernatant at a specific time *t* > 0.

### 2.5. Cell Culture

The T24 cells (EGFR-expressing human bladder carcinoma cell line; ATCC, Rockville, MD, USA) were cultured in McCoy’s 5a medium supplemented with 10% FBS, 1.5 mM l-glutamine, and 100 U mL^−1^ penicillin-streptomycin, and were maintained in a 37 °C incubator that was balanced with 5% CO_2_ and 100% humidity.

### 2.6. Verification of EGFR Binding Specificity of the EIMPDNEs

The binding specificity of the EIMPDNEs to EGFR-expressing bladder cancer cells was determined by assessing the adsorption efficiency of the EIMPDNEs in the T24 cells with and without binding-competitive molecules. Briefly, one day before the experiment, 3 × 10^6^ T24 cells were aliquoted into six wells of a 24-well culture plate and were incubated at 37 °C until the time of use. For the non-competitive experimental setting, EIMPDNEs and IMPDNEs that each contained 20 µM ICG and 18 µM MMC were separately added to one of the six wells and incubated at 37 °C for 4 h. For the EGFR competitive experimental setting, the cells in another three of the six wells were first treated with 0.5, 1, and 2 μg mL^−1^ of naked anti-EGFR-mAb separately at 37 °C for 4 h, followed by co-culture with EIMPDNEs for an additional 4 h. After being washed twice with PBS, the intensity of ICG-derived fluorescence expressed from each group of cells was measured by using a spectrofluorometer with a 750 nm excitation wavelength and 838 nm emission wavelength, and was quantitatively represented by the RFUs. In this study, the group without nanodroplets was employed as the control. The cell binding efficiency of the EIMPDNEs was analyzed using the RFUs after normalization against the control.

### 2.7. Assessment of the EIMPDNE-Induced Hyperthermia Effect

To evaluate the hyperthermia effect of the EIMPDNEs upon NIR illumination, 200 µL of DI water containing EIMPDNEs or free ICG molecules (i.e., ICG solution) with 0, 2, 4, 20, 40, and 100 µM of ICG concentrations were separately exposed to an 808 nm laser, with an output intensity of 6 W cm^−2^ in one well of a 96-well culture plate. The temperature of each group was detected using a digital thermometer (TES-1316, Instrumentation Sales and Rentals, Tonawanda, NY, USA) every 30 s for 5 min. 

### 2.8. Assessment of Singlet Oxygen Production Induced by EIMPDNEs

To evaluate the photodynamic efficacy of the EIMPDNEs upon NIR illumination, the production of singlet oxygen generated from the EIMPDNEs or freely dissolved ICG with 0, 2, 4, 20, 40, and 100 µM of ICG concentration under NIR exposure was separately measured using the singlet oxygen sensor green (SOSG) kit (Life Technologies, Carlsbad, CA, USA). The NIR treatment was conducted by using an 808 nm laser with a 6 W cm^−2^ output intensity throughout the experiment. The level of SOSG-induced fluorescence in each group was detected by using a spectrofluorometer (Synergy^TM^ HT, BioTek, Winooski, VT, USA) with an excitation wavelength of 488 nm and an emission wavelength of 525 nm every 60 s for 5 min, and was quantitatively represented by the RFUs. 

### 2.9. In Vitro Cytotoxicity Assay

To evaluate the photochemotoxicity of the EIMPDNEs, the T24 cells were plated in 22 wells of a 96-well culture plate with 1 × 10^5^ cells/well for 24 h. Afterward, the cells were separately treated with fresh medium ± NIR, free ICG + NIR, free MMC, or EIMPDNEs ± NIR, each using different procedures. For the groups with the NIR treatment, the cells were washed twice with PBS after a 4 h incubation with a blank medium, free ICG, or EIMPDNEs, followed by NIR exposure (808 nm; 6 W cm^−2^) for 5 min. The cells were then incubated at 37 °C for an additional 24 h and subjected to viability analyses thereafter. For the groups without NIR treatment, the viabilities of the cells were directly measured after incubation with or without agents for 24 h. In this study, the concentrations of free ICG and MMC were determined based on the dosages provided by the EIMPDNEs, and those were 2, 4, 20, 40, and 100 µM for ICG and 1.8, 3.6, 18, 36, and 90 µM for MMC, respectively. Both hemocytometry and calcein-AM/propidium iodide (PI) staining assays were applied as a part of the cell viability measurements.

### 2.10. Statistical Analysis

All data were acquired from three independent experiments and are presented as the mean ± standard deviation (s.d.). Statistical analyses were conducted using MedCalc software (Ostend, Belgium) in which comparisons for one condition between two groups were performed by using one-way analyses of variance (ANOVA) with a significance level of *p* < 0.05 throughout the study.

## 3. Results and Discussion

### 3.1. Characterization of EIMPDNEs

**Figure 2:**
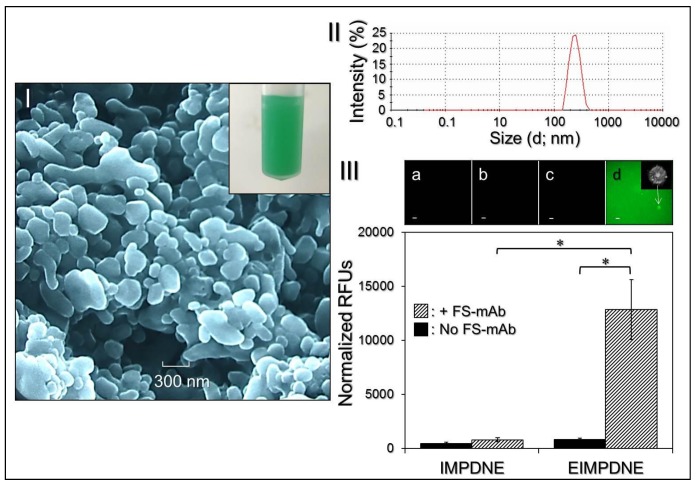
Assessment of physical and chemical properties of the EIMPDNEs. (**I**) SEM image of the EIMPDNEs detected at 10,000× magnification. The inset photograph shows the appearance of the EIMPDNE sample. (**II**) Size distribution profile of the EIMPDNEs measured by DLS technique. (**III**) Validation of the presence and bioactivity of anti-EGFR-mAbs on the EIMPDNE surface. The upper photographs are the representative fluorescence microscopic images of the IMPDNEs (**a**,**b**) and EIMPDNEs (**c**,**d**) without (**a**,**c**) and with (**b**,**d**) FS-mAb conjugation at 200× magnification. Scale bar = 5 μm. The inset photomicrographic image in Figure 2IIId represents an EIMPDNE under optical microscopy at 400× magnification. The fluorescence level expressed from each group was detected using a fluorospectrometer set at an excitation wavelength of 488 nm and emission wavelength of 525 nm, and was quantitatively represented and analyzed by relative fluorescence units (RFUs) after normalization against the background signal. Values are mean ± s.d. (*n* = 3). * *p* < 0.05.

Figure 2I shows the appearance of the EIMPDNE sample (inset photograph) and the SEM image of the EIMPDNE particles, where the intact particulate morphology indicates that the EIMPDNEs were able to maintain their structure without disintegration after the manufacturing processes—including filtration, high-speed centrifugation, and agitation. Based on the DLS analyses, the surface charge of the EIMPDNEs was approximately −12.3 ± 2.33 mV, whereas the nanodroplet size was about 257 ± 19.4 nm with a polydispersity index of 0.07–0.18 after filtration (Figure 2II). From the calculations of Equations (1) and (2), the encapsulation rates of ICG and MMC are 96.2 ± 1.73% and 41.7 ± 6.86%, respectively, whereas the loading rates of ICG and MMC in the EIMPDNE are about 0.3 ± 0.02 wt % and 0.1 ± 0.05 wt %, respectively.

We subsequently examined the bioactivity of the conjugated anti-EGFR-mAb while using the secondary antibody assay. The EIMPDNEs displayed significant fluorescence expression after treatment with the FS-mAb (Figure 2III, upper image d) and the detected fluorescence magnitude was 16-fold (*p* < 0.05) higher than that gained from the EIMPDNEs without FS-mAb (Figure 2III, upper image c). Moreover, the level of fluorescence expressed from the EIMPDNEs with FS-mAb was 16.4-fold (*p* < 0.05) higher than that obtained from the FS-mAb-treated IMPDNE (Figure 2III, inset image b), demonstrating that the enhanced fluorescence expression in the former group can certainly be attributed to the first-secondary antibody conjugation instead of FS-mAb adsorption on the particle surface due to electrostatic interaction and/or an imperfect wash. These results clearly show that the EIMPDNEs have an affinity to the corresponding secondary antibody molecules, indicating that the anti-EGFR-mAbs were anchored on the EIMPDNE surface and they were able to provide an intact bioconjugation activity after the carboxyl-amine crosslinking reaction. In addition, one of micron-scaled FS-mAb-conjugated EIMPDNEs was accidentally caught, as illustrated in Figure 2II (inset photograph in upper image d), where the double-layer structure of the nanodroplet can be clearly observed.

### 3.2. Thermal Stability of EIMPDNE-Entrapped ICG and Efficiency of MMC Release

**Figure 3:**
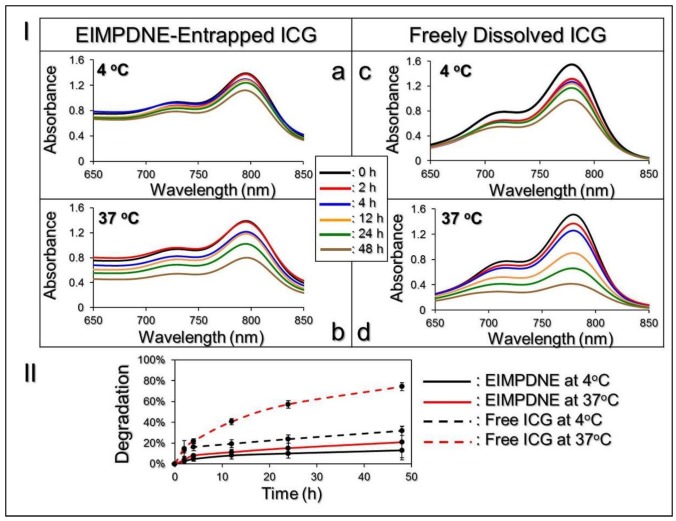
Evaluation of thermal stability of the EIMPDNE-entrapped ICG in comparison with freely dissolved ICG in deionized (DI) water. (**I**) **a**–**d** show the UV-Vis spectra of EIMPDNE-entrapped ICG (**a**,**b**) and free indocyanine green (ICG) (**c**,**d**) in DI water under 4 (**a**,**c**) or 37 °C (**b**,**d**) incubation in the dark for 0, 2, 4, 12, 24, and 48 h. The absorbance value detected at λ = 780 nm in each spectrum curve represents the level of intact ICG remaining in the sample at the time of measurement. (**II**) Degradation curves of the EIMPDNE-entrapped ICG and freely dissolved ICG in DI water that was maintained at 4 or 37 °C for 48 h. Values are mean ± s.d. (*n* = 3).

Figure 3I exhibits the degradation profiles of the EIMPDNE-entrapped ICG (Figure 3Ia,b and freely dissolved ICG in DI water (Figure 3Ic,d) under incubation at 4 or 37 °C in the dark for 48 h. Based on the absorbance value analyses at λ = 780 nm (Figure 3II), the results showed that the degradation rates of ICG in the EIMPDNEs were 2.4 and 3.6-fold lower than that in the DI water within 48 h at 4 and 37 °C, respectively.

Moreover, based on the *k*_d_ analyses, as shown in Table 1, the anti-degradability of the EIMPDNE-entrapped ICG at 4 and 37 °C was significantly enhanced by 2.8 (*p* < 0.05) and 5.8 (*p* < 0.05)-fold, respectively, when compared to freely dissolved ICG under equal temperature treatment for 48 h. These results indicate that the thermal stability of ICG was significantly enhanced after being encapsulated in the EIMPDNE.

**Figure 4:**
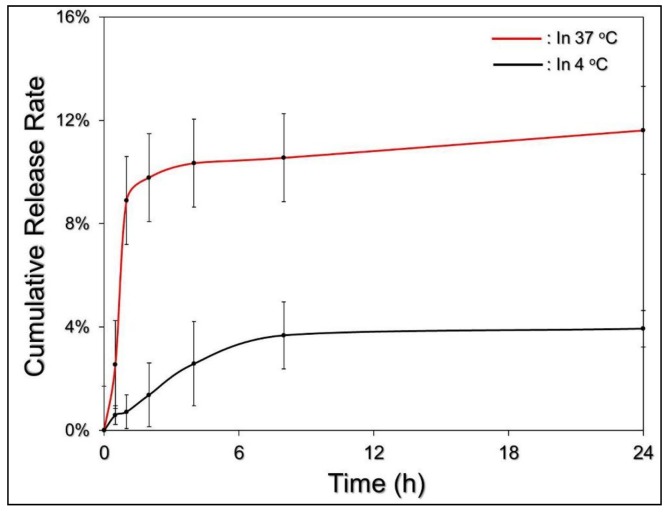
The MMC release profiles of the EIMPDNEs under different temperature settings. The cumulative release curves of the EIMPDNE-entrapped MMC under a maintained temperature of 4 and 37 °C were plotted through measurement of the MMC concentration in the supernatant using spectrophotometry at λ = 485 nm after treatment for 0, 0.5, 1, 2, 4, 8, and 24 h. Values are mean ± s.d. (*n* = 3).

Figure 4 shows the cumulative release profiles of the EIMPDNE-entrapped MMC at 4 or 37 °C. Both of the groups exhibited a biphasic drug release profile that was consistent with findings from a number of studies [36,37], and the overall release rates after incubation at 4 and 37 °C for 24 h were 3.94 ± 0.71% and 11.61 ± 2.88%, respectively. We reason that the markedly low MMC release rate of EIMPDNE in 4 °C was mainly attributed to the exceptional chemical and thermal stability of the entrapped PFC (PFOB), due to its high strength of the carbon-fluorine bond (ca. 487 kJ mol^−1^) and the robust steric and electronic protection that is provided by the fluorine atoms [33]. In addition, we surmise that the burst release of the encapsulated MMC in the first few minutes of heating at 37 °C resulted from demulsification (i.e., phase inversion/separation, coalescence, and/or Ostwald ripening) of the EIMPDNEs because an increased temperature may be able to reduce the emulsion viscosity and facilitate Brownian motion of the nanoparticles [38]. Therefore, the collision rate of the nanodroplets increased and led to a rapid MMC release. The change of the emulsion configuration may subsequently reach an equilibrium state, and hence the drug release rate was decreased. Moreover, in comparison with other, similar products reported previously, the release efficiency of the MMC from the EIMPDNEs is lower than that released from the liposome [39], micelle [40], and polymeric [41] nanostructures, implying that the MMC is relatively stable in the EIMPDNEs. We speculate that the moderated drug release rate of the EIMPDNE can be attributed to (1) less reactivity of the nanodroplets because the electrostatic repulsion generated from the surface charge may diminish their interactions with foreign molecules and/or each other, conferring an enhanced shelf stability to the EIMPDNEs in an aqueous medium, and (2) a higher degree of steric hindrance on the emulsion surface that is caused by a tangled PEO-PPO-PEO copolymer and/or antibody molecules. 

### 3.3. Binding Specificity of EIMPDNEs

**Figure 5:**
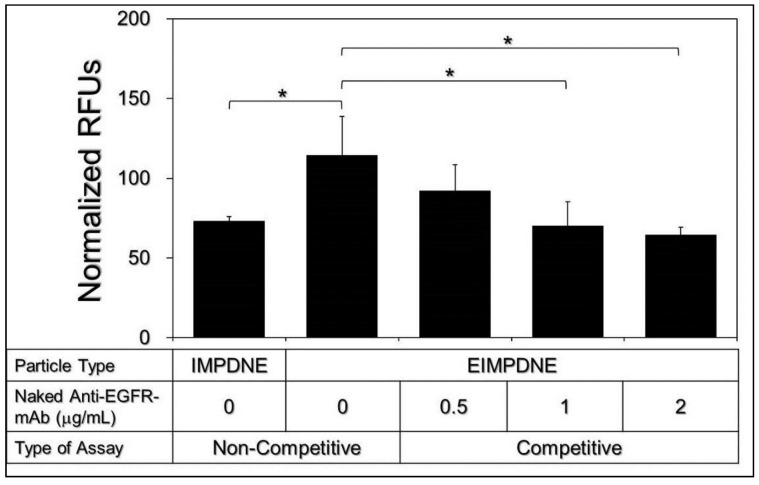
Identification of the EGFR-binding specificity of the EIMPDNEs in vitro using EGFR-expressing T24 cells. In the non-competitive assay, the cells were treated with IMPDNEs or EIMPDNEs in the absence of anti-EGFR-mAb for 4 h. Both types of nanodroplets were used in a 20 µM ICG/18 µM MMC equivalent concentration to the cells. For the competitive assay, the cells were first co-incubated with 0.5, 1, or 2 μg mL^−1^ of naked anti-EGFR-mAb at 37 °C for 4 h, followed by incubation with EIMPDNEs for another 4 h. The ICG-derived fluorescence in each group was detected using spectrofluorometry performed with a 750 nm excitation wavelength and 838 nm emission wavelength immediately after the nanodroplets were removed, and they were quantitatively analyzed by RFUs after normalization against the group without nanodroplets. Values are mean ± s.d. (*n* = 3), * *p* < 0.05.

Figure 5 shows the levels of ICG-induced fluorescence expressed from the T24 cells after treatment with IMPDNEs or EIMPDNEs with and without anti-EGFR-mAb competition for 4 h. 

In terms of the non-competitive study, the normalized fluorescence level detected from the EIMPDNE-treated cells was 1.6-fold (*p* < 0.05) higher than that obtained from the cells with IMPDNEs, indicating that the amount of the EIMPDNE on the T24 cell membrane was significantly higher than those treated with IMPDNEs. To ensure that the increased adhesion of the EIMPDNE on the EGFR-expressing cells (T24 cells) was due to conjugation with cellular EGFR receptors, the binding effect of the EIMPDNEs to the T24 cells in the presence of anti-EGFR-mAb molecules was further investigated. As shown in Figure 5, the ICG-derived fluorescence level expressed from the cells was significantly decreased to 45% (*p* < 0.05) when the concentration of the competitive molecule (i.e., free anti-EGFR-mAb) was increased from 0 to 2 μg mL^−1^. As to the question of why the two types of nanodroplets exhibited different cell binding efficacies, we speculate that it was because the uptake of the EIMPDNEs in the EGFR-expressing T24 cells was conducted through EGFR receptor-mediated endocytosis, while IMPDNE internalization was carried out through an adsorptive endocytosis—which is an efficient mechanism for cancer cells to internalize negatively charged nanoparticles, as reported in a previous study [42]. Since receptor-mediated endocytosis is more specific and efficient than adsorptive endocytosis [43], it is reasonable that we found that the cells with EIMPDNEs were able to display higher RFUs when compared to the group with IMPDNEs.

### 3.4. Effects of Hyperthermia and Singlet Oxygen Generation of EIMPDNEs

**Figure 6:**
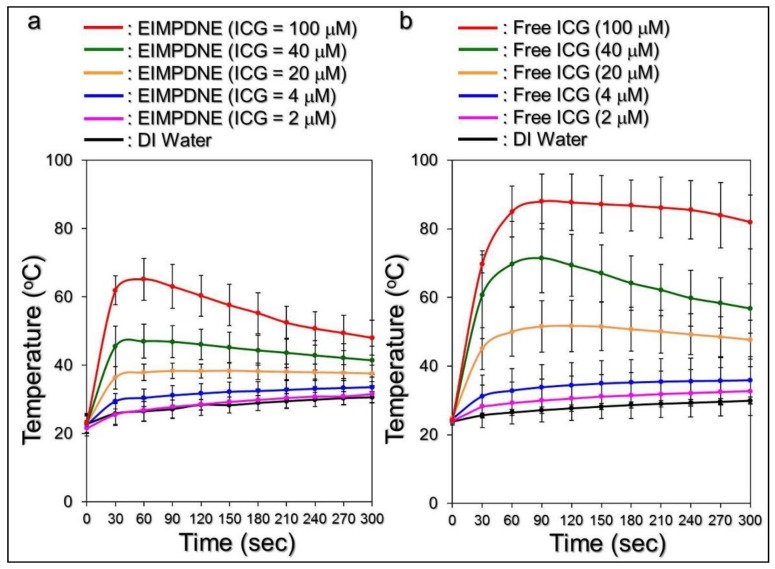
Assessments of EIMPDNE and freely dissolved ICG-induced hyperthermia effects under light illumination. Upon near infrared (NIR) laser irradiation (808 nm; 6 W cm^−2^), the temperatures in the samples of EIMPDNEs (**a**) and freely dissolved ICG (**b**) with equal ICG concentrations of 0 (DI water only), 2, 4, 20, 40, and 100 μM were separately measured using a digital thermometer every 30 s for 5 min. Values are mean ± s.d. (*n* = 3).

Figure 6 shows the hyperthermia effects that were generated from the various concentrations of EIMPDNEs (Figure 6a) and freely dissolved ICG (Figure 6b) under NIR treatment (808 nm; 6 W cm^−2^) for 5 min. Similarly to the results that were achieved by free ICG, the temperature in each EIMPDNE group quickly elevated within the first minute of NIR treatment and then was sustained at approximately the same level (groups with ≤20 µM ICG) or slowly declined (groups with ≥40 µM ICG) afterward, yielding an increase of 7.8, 10, 11.6, 14.7, 18.5, and 25.2 °C for the EIMPDNEs with 0 (DI water only), 2, 4, 20, 40, and 100 µM ICG, respectively, after 5 min of NIR exposure.

However, one may notice that the ultimate temperature level in each EIMPDNE group was lower than the value obtained from the freely dissolved ICG with an equal ICG concentration. We speculate that this was because, in contrast with the ICG solution, all of the free ICG molecules were able to simultaneously react upon NIR exposure and the hyperthermia effect of the EIMPDNEs was achieved by partially released ICG. Furthermore, demulsification that is caused by NIR irradiation is a process of heat absorption [44] that may diminish the thermal energy received by the medium. Therefore, the level of the EIMPDNE-induced thermal effect was relatively moderate when compared to that triggered by freely dissolved ICG, as shown in Figure 6. Nonetheless, these outcomes clearly demonstrate that the EIMPDNEs are able to provide a dose-dependent hyperthermia effect upon NIR treatment.

**Figure 7:**
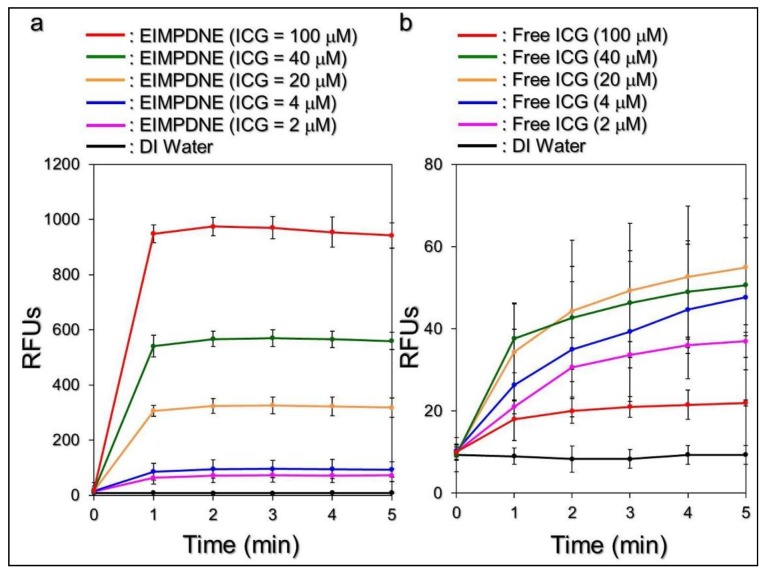
Assessments of EIMPDNE and freely dissolved ICG-induced singlet oxygen productions under light illumination. Upon NIR laser exposure (808 nm; 6 W cm^−2^), the yields of singlet oxygen generated from the EIMPDNEs (**a**) and freely dissolved ICG (**b**) with equal ICG concentrations of 0 (DI water only), 2, 4, 20, 40, and 100 μM were separately measured every 60 s for 5 min. The amount of singlet oxygen produced in each group was evaluated based on the intensity of SOSG-induced fluorescence detected using a spectrofluorometer with a 488 nm excitation wavelength and 525 nm emission wavelength, and was represented by RFUs. Values are mean ± s.d. (*n* = 3).

Figure 7 shows the effects of singlet oxygen production yielded from various concentrations of the EIMPDNE (Figure 7a) or freely dissolved ICG (Figure 7b) with a 5 min NIR exposure. 

Our data show that the EIMPDNEs with ≤100 µM of ICG enabled a dose-dependent production of singlet oxygen throughout the dose range of 0–100 µM of ICG, as performed by the free ICG with ≤20 µM. However, the yields of singlet oxygen generated from the free ICG with ≥40 µM were inversely correlated to the ICG concentration, as plotted in Figure 7b. Such a decrease of singlet oxygen generation at a high concentration of ICG is reasoned to be due to the aggregation behavior of ICG, as reported previously [45]. Furthermore, it can be seen that the EIMPDNE-induced singlet oxygen productivity was exceptionally higher than that obtained from the same concentration of free ICG. According to the RFU analysis, the yields of singlet oxygen that were generated from the EIMPDNEs were 2, 2.5, 5.8, 11.2, and 42.8-fold (*p* < 0.05 for each) higher than that induced by the freely dissolved ICG when the concentrations of ICG in both groups were set as 2, 4, 20, 40, and 100 µM, respectively. These results clearly show that the EIMPDNEs were able to provide an enhanced amount of singlet oxygen compared to the same concentration of free ICG upon NIR laser exposure, and this improved efficacy was attributed to the PFC (PFOB) that possesses high oxygen dissolubility in the EIMPDNEs.

It has been previously found that the primary cause of cancer cell death during thermal ablation is acute coagulative necrosis, in which the temperature level plays the key role in the efficacy of thermal therapy (e.g., PTT). According to previous studies, irreversible cell injury can be obtained after heating the cells at 40–45 °C for 30–60 min [46]. Up to 50–52 °C, irreversible cell damage is intensified, and just 4–6 min is sufficient to cause necrosis for a significant population of cells [47]. At temperatures in excess of 60 °C, the time required to cause irreversible cell death further decreases because such high temperatures may cause rapid denaturation of cytoplasmic proteins or enzymes and serious melting of the plasma membrane, leading to immediate necrosis [48]. Although an elevated temperature may offer a higher efficacy of tumor eradication, a moderate temperature setting of 41–43 °C is more frequently used in the clinic to avoid any possible heating-induced adverse effects, such as water vaporization, desiccation, and/or carbonization in the surrounding normal cells and tissues [49]. In this study, we speculate that EIMPDNEs with ≥40 μM ICG are able to provide both photothermal (*T* > 41 °C) and photodynamic effects for cancer cell eradication under NIR exposure (808 nm; 6 W cm^−2^), while those with ≤20 µM ICG are solely photodynamic in function, based on the results shown in Figure 6 and Figure 7.

### 3.5. Efficiency of MMC Release under NIR Exposure

**Figure 8:**
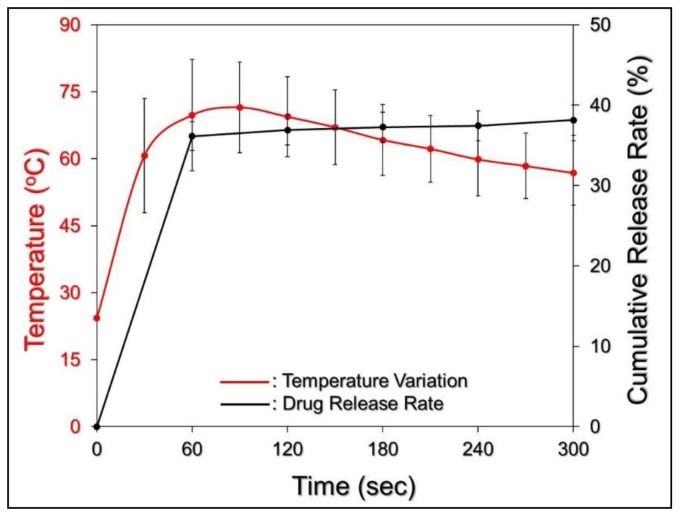
The MMC release profile of the EIMPDNEs under NIR illumination in vitro. The black curve represents the cumulative release profile of the EIMPDNE-entrapped MMC (black) under NIR exposure (808 nm; 6 W cm^−2^) and was plotted by measuring the concentrations of MMC in the bulk phase through spectrophotometry at λ = 485 nm after treatment for 0, 1, 2, 3, 4, and 5 min. The red curve indicates the temperature variation of the EIMPDNE-containing medium with 40 μM of ICG equivalent concentration under NIR exposure for 5 min. Values are mean ± s.d. (*n* = 3).

The efficiency of MMC release under NIR exposure (808 nm; 6 W cm^−2^) was subsequently examined by using EIMPDNEs with 40 μM ICG/36 μM MMC. As shown in Figure 8, a two-phase MMC release profile was obtained, as it was given in the absence of NIR irradiation (Figure 4), and the cumulative release rate was 38.5 ± 2.6% after a 5 min NIR treatment. 

The temperature of the EIMPDNE sample was able to achieve >70 °C within 60 s of NIR treatment, and this is much higher than the melting temperature of the Pluronic F68 (*T*_m_ = ~54 °C) [50]. This is the polymer that is used to build up the outer layer of the EIMPDNE. It is therefore reasonable to conclude that the collapse of the EIMPDNE occurred shortly after the NIR treatment, and it led to a rapid release of MMC from the nanodroplets in the first minute of light illumination. In comparison with the results shown in Figure 4, here our data clearly indicate that the efficiency of MMC release from the EIMPDNEs can be promoted through NIR irradiation. 

### 3.6. In Vitro Cytotoxicity of EIMPDNEs

**Figure 9:**
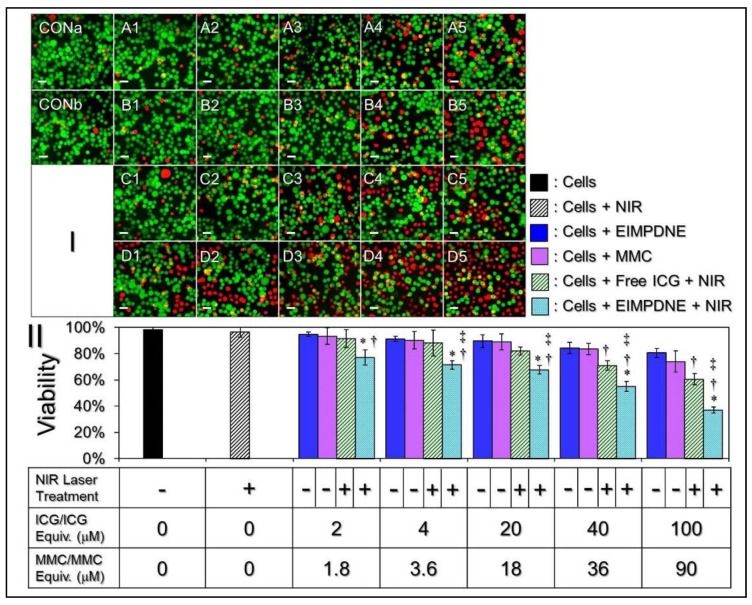
Cytotoxicity of the EIMPDNEs to EGFR(+) bladder cancer cells in vitro. (**I**) Photomicrographic images of T24 cells under different treatments. The rows A and B represent the groups in which the cells were co-cultured with the EIMPDNEs (**A**) or free mitomycin C (MMC) (**B**) for 24 h. The rows **C** and **D** represent the groups in which cells were pre-co-cultured with free ICG (**C**) or EIMPDNEs (**D**) for 4 h, followed by treatment with NIR irradiation (808 nm; 6 W cm^−2^) for 5 min and incubation at 37 °C for an additional 24 h. The columns 1–5 denote that the cells were treated with free ICG in 2, 4, 20, 40, or 100 μM doses, respectively (Row **C**), free MMC in 1.8, 3.6, 18, 36, or 90 μM doses, respectively (Row **B**), or EIMPDNEs with combined ICG/MMC in 2/1.8, 4/3.6, 20/18, 40/36, or 100/90 μM doses, respectively (Rows **A**,**D**). CONa denotes the cells with neither compound (ICG and/or MMC) treatment nor NIR exposure. CONb represents the cells treated with NIR irradiation for 5 min followed by incubation at 37 °C for 24 h. The green and red cells represent live and dead cells, respectively, formed by calcein-AM/PI staining. All images were photographed by fluorescence microscopy at 200× magnification. Scale bar = 30 μm. (**II**) Quantitative analyses of the viabilities of T24 cells after treatment with free MMC, free ICG, or EIMPDNEs under various dosages, as indicated on the *X* axis. Values are mean ± s.d. (*n* = 3). * *p* < 0.05 compared to the EIMPDNE-treated group without NIR illumination. ^†^ *p* < 0.05 when compared to the group with an equal dose of free MMC. ^‡^ *p* < 0.05 as compared to the group with an equal dose of free ICG and NIR treatment.

Figure 9 shows the viability rates of T24 cells after treatments with various doses of ICG, MMC, and/or EIMPDNEs, with and without NIR laser exposure (808 nm, 6 W cm^−2^). Based on the hemocytometric analyses, as plotted in Figure 9II, the viability of the cells with NIR alone (Figure 9I, CONb) was 96.5%, showing that the medium with a slightly increased temperature due to NIR exposure (Figure 6) was nontoxic. On the other hand, it can be seen that the cytotoxicity in each experimental group (Figure 9I; row A–D) was increased along with an increase of drug dosage, and the results show that the cells treated with the EIMPDNEs in association with NIR exposure (Figure 9I, row D) exhibited a higher mortality rate when compared with the cells that were treated by either (1) EIMPDNEs without NIR exposure (Figure 9I, row A), (2) MMC alone (Figure 9I, row B), and (3) free ICG + NIR irradiation (Figure 9I, row C) (*p* < 0.05 for all of the comparisons when the dose of the EIMPDNE reached ≥4 µM ICG/3.6 µM MMC). Viabilities of 77.1%, 71.4%, 67.7%, 55.1%, and 43.2% were obtained when the concentrations of ICG/MMC provided by the EIMPDNEs were set as 2/1.8, 4/3.6, 20/18, 40/36, and 100/90 µM, respectively. These outcomes clearly show that the EIMPDNEs are able to achieve effective bladder cancer cell eradication upon NIR irradiation (808 nm; 6 W cm^−2^), but are less toxic in the absence of light illumination. 

To minimize the potential chemodrug-induced side effects, in this study, the EIMPDNEs were used with up to 90 µM of MMC. This is much lower than the dosage typically used in the clinic (>1 mM) [51,52,53]. However, a robust cytotoxicity of the EIMPDNE upon NIR irradiation can still be obtained in each setting, and the resulting mortality rate was even higher than that caused by using a five-fold increased amount of free MMC alone (Figure 9II). These outcomes imply that phototherapy played a crucial role in the EIMPDNE-mediated anticancer treatment. Moreover, the significance of phototherapy can also be demonstrated through comparing the viability of cells that were treated with free ICG + NIR (Figure 9I; row C) to those that were stimulated by free MMC alone (Figure 9I; row B). Our data show that the mortality rates of the cells with 2, 4, 20, 40, and 100 µM free ICG under NIR exposure markedly increased by 2%, 3%, 7%, 12% (*p* < 0.05), and 15% (*p* < 0.05) when compared with the mortality rate that was obtained from the groups with 1.8, 3.6, 18, 36, and 90 µM of MMC alone (Figure 9II). This indicates that phototherapy indeed played a predominant role in EIMPDNE-mediated cancer cell eradication. However, the MMC-mediated chemotherapeutics from the EIMPDNEs are indispensable because they may take over the therapeutic role from ICG after NIR treatment, and provide a relatively long-term anticancer effect thereafter. To further enhance the anticancer efficacy of the EIMPDNEs, the use of other cell surface antigens, or a cocktail of several different chemo-drugs and/or photosensitizers in the payload may be a useful strategy, however these procedures will certainly need to be examined through experiments to confirm their efficacy. Taken all together, with the merits of an improved ICG stability, EGFR binding specificity, and robust efficacy of cancer cell eradication, EIMPDNE is considered to be a more beneficial photosensitizer than free ICG in terms of use for phototherapy, and it is anticipated to be able to cause less chemotoxicity in cancer treatment due to its decreased chemo-dosage in the payload, making it highly advantageous for use in the clinic. 

## 4. Conclusions

Due to the prevalence of EGFR expression in bladder cancer cells, EGFR-target noninvasive therapeutics have been recognized as a viable approach for treatment, and consequently have been widely studied in the past decade. In this paper, we have presented a proof-of-concept study of targeted photochemotherapeutics for EGFR-expressing bladder cancer cells using developed EIMPDNEs. We not only investigated the nanodroplets’ physicochemical properties and functionalities, but also evaluated their effectiveness in an anticancer application in vitro. In addition to the aforementioned advantages of the EIMPDNE, the nonionic PEO/PPO/PEO block copolymer on the outer surface of the nanodroplet could diminish the P-glycoprotein activity of the drug-resistant cells by reducing their ATP productivity [54], and this may allow for us to avoid the serious drug resistance frequently occurring in conventional EGFR inhibition approaches [55]. Moreover, the mild negative change (−12.3 ± 2.33 mV) of the EIMPDNE offers a proper surface ζ-potential for cancer cell internalization since it may support more efficient accumulation at the tumor site when compared with highly negative or positively charged particles, as reported in the previous study [56]. On the other hand, since (1) there are conflicting data on EGFR expression in UCC and normal bladder mucosa and (2) EGFR expression in normal bladder cells may possibly increase side effect of EIMPDNE-mediated treatment, the PFC double emulsion-based drug carrier can be decorated by other cell surface proteins that are overexpressed or aberrantly expressed in bladder cancer cells, such as FGFR-3, ErbB-2, Nectin-4, Muc-1, CEA, and so forth, in order to improve the availability or clinical utility of the nanodroplet. However, further studies are needed to explore this idea. Overall, given the high amplification rate of EGFR in most bladder cancer cells, EIMPDNEs may provide a selective and improved therapy for NMBIC. Currently, we are actively conducting orthotopic murine models to examine the effect of EIMPDNEs in vivo and aim to translate our efforts into a viable clinical strategy for bladder cancer patients in the future.

## Figures and Tables

**Table 1:** Analyses of residual percentages and degradation rate coefficients of the EIMPDNE-entrapped ICG and freely-dissolved ICG after a 48 h treatment.

Group/Temperature Setting	Residual Rate of ICG (*C*_t _ */*C*_0_)	*k*_d_ (h^−1^)
EIMPDNE-entrapped ICG		
4 °C in the dark	86.90%	0.0029 ^†^
37 °C in the dark	79.08%	0.0049 ^†^
Free ICG in DI Water		
4 °C in the dark	68.07%	0.0081
37 °C in the dark	25.62%	0.0284

* *C*_t_ = Concentration of ICG in the EIMPDNEs or in DI water after 48 h incubation at 4 or 37 °C. ^†^ *p* < 0.05 compared to the value gained from the group with free ICG under equal temperature settings.
